# Triglyceride glucose index exacerbates the risk of future cardiovascular disease due to diabetes: evidence from the China Health and Retirement Longitudinal Survey (CHARLS)

**DOI:** 10.1186/s12872-022-02673-y

**Published:** 2022-05-21

**Authors:** Zixiang Ye, Enmin Xie, Siqi Jiao, Yanxiang Gao, Peizhao Li, Yimin Tu, Ziyu Guo, Qing Li, Yaxin Wu, Xiaozhai Yu, Yike Li, Changan Yu, Jingyi Ren, Jingang Zheng

**Affiliations:** 1 Department of Cardiology, Peking University China-Japan Friendship School of Clinical Medicine, Beijing, 100029 China; 2 Graduate School of Peking Union Medical College, Chinese Academy of Medical Sciences and Peking Union Medical College, Beijing, 100029 China; 3 Department of Cardiology, China-Japan Friendship Hospital, Beijing, 100029 China

**Keywords:** Triglyceride glucose index, Diabetes, Cardiovascular disease, Adults, Chinese

## Abstract

**Objective:**

We aimed to investigate the effect of the triglyceride glucose (TyG) index on the association between diabetes and cardiovascular disease (CVD).

**Methods:**

Data from 6,114 individuals were extracted and analyzed from the China Health and Retirement Longitudinal Survey (CHARLS) from 2011 to 2018. Logistic regression analyses were conducted to assess the relationship between diabetes and CVD across the various TyG index groups. The statistical method of subgroup analysis was used to determine the correlation between diabetes and CVD for each TyG index group by sex, history of hypertension and dyslipidemia, smoking, and drinking.

**Results:**

Diabetes was positively associated with CVD risk after adjustment in 2011(odds ratio (OR) 1.475, 95% CI 1.243–1.752, *P* < 0.001). There was a gradient increase in the OR for new-onset CVD in 2018 due to diabetes at baseline across the value of the TyG index based on a fully adjusted model (*P for* trend < 0.05). The ORs of diabetes at baseline for CVD in 2018 were 1.657 (95% CI 0.928–2.983, *P* = 0.098), 1.834(95% CI 1.064–3.188, *P* = 0.037) and 2.234(95% CI 1.349–3.673, *P* = 0.006) for T1, T2 and T3 of the TyG index respectively. The gradient of increasing risk of CVD still existed among those with hypertension and nondrinkers in the subgroup analysis.

**Conclusion:**

Elevated TyG index strengthens the correlation between diabetes mellitus and CVD in middle-aged and elderly Chinese adults.

## Introduction

According to the International Diabetes Federation, the prevalence of diabetes will likely increase globally from 9.3% (463 million people) in 2019 to 10.9% (700 million) in 2045 [1]. The risk of developing cardiovascular disease (CVD) has been reported to be approximately twofold higher in people with diabetes, in whom CVD is the primary cause of death [2, 3]. Therefore, better management must identify CVD development in patients with diabetes.

Insulin resistance (IR) is an abnormal metabolic state in which the target tissue has a lower response to the average insulin concentration [4, 5] and has been proposed as the critical linking factor for diabetes and cardiovascular diseases [6]. Currently, the hyperinsulinemic-euglycemic clamp is regarded as the gold standard for evaluating IR [7, 8]. However, because of the tedious and labor-intensive procedure needed, the euglycemic hyperinsulinemic clamp technique is impractical for measuring insulin sensitivity directly in large-scale epidemiological studies with limited clinical value.

The triglyceride glucose (TyG) index is a reliable alternate of IR [9]. Many studies in various countries have demonstrated that the TyG index is a crucial predictor for diabetes development [10]. Additionally, several researchers have reported a positive association between an elevated TyG index and vascular disease, including carotid atherosclerosis, arterial stiffness, and major adverse cardiovascular events (MACEs) [11–14]. The TyG index was also positively correlated with the severity of CVD and cardiovascular outcomes [15]. Unfortunately, few studies have investigated cardiovascular effects on individuals with diabetes of various TyG index levels and the interaction of the TyG index between CVD and diabetes.

In this study, a large-scale population dataset from the China Health and Retirement Longitudinal Study (CHARLS) was used to estimate whether the value of the TyG index could impact the association between diabetes and future cardiovascular disease.

## Methods

### Study design and population

**Fig. 1:**
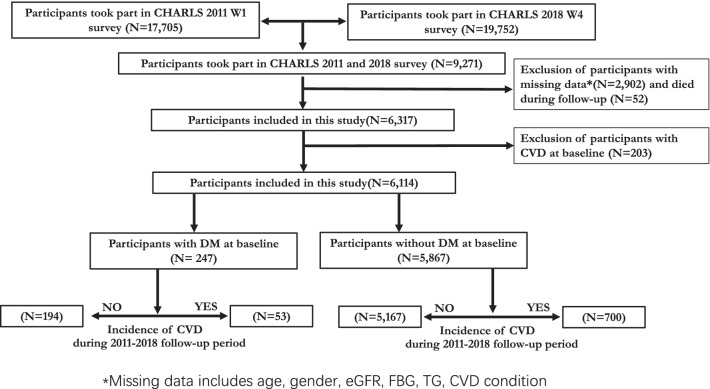
Flowchart of the procedure extracting the patients from CHARLS

The data for this cohort study were extracted from CHARLS, a large-scale longitudinal prospective cohort study in China enrolling adults aged 45 years or above. The objective, study design and methods of CHARLS have been described in detail elsewhere [16, 17]. In short, the CHARLS recruited 17,708 participants within 28 provinces in China, adopting a multistage probability sampling technology in the first wave (W1) between June 2011 and March 2012. All participants were Chinese, with an 81% response rate to the W1 survey. The 9,271 individuals with available baseline data from W1 and follow-up data from the fourth wave (W4, 2018) were enrolled in this study. Participants with missing data of blood tests on triglycerides (TG), fast blood glucose (FBG), and self-reported history of CVD and diabetes were excluded. Finally, after further excluding 203 participants with CVD at W1, 6,114 individuals were recruited for this study (Fig. 1). All methods were carried out in accordance with relevant guidelines and regulations. The ethical approval and experimental protocols were approved by the institutional review board of Peking University (IRB00001052–11,015). Informed consent was acquired from all participants before participation.

### Data collection and definition

At baseline, trained researchers used a structured questionnaire to acquire sociodemographic status information and health-related indicators, including age, gender, educational level, smoking and drinking status. Education level was stratified as primary school or lower, junior high school and senior high school or above. Fundamental health indicators included self-reported drinking and smoking status (never, former, or current), medical condition (self-reported physician diagnosis of CVD, hypertension, diabetes, and dyslipidemia) and medication treatment for CVD, diabetes, hypertension, and dyslipidemia. Trained nurses were responsible for measuring height, weight, and diastolic and systolic blood pressure. Venous blood samples were gathered and determined for high-sensitivity C-reactive protein (hsCRP), glucose, blood urea nitrogen (BUN), cystatin C, uric acid, total cholesterol (TC), high-density lipoprotein cholesterol (HDL-C), low-density lipoprotein cholesterol (LDL-C), TG and creatinine by the staff of the Chinese Center for Disease Control and Prevention.

The Incidence of developing CVD events was defined as the positive answer to the question of whether the patient had doctor-diagnosed heart disease (including heart attack, coronary heart disease, angina, congestive heart failure, or other heart problems) or stroke during the follow-up period, which was regarded as the outcome of this research. Diabetes was defined as a self-reported doctor’s diagnosis of diabetes, taking treatment of antidiabetic medications, FBG > 125 mg/dl or HbA1C > 6.5% in the blood test. The equation ln[TG(mg/dl) *FBG(mg/dl) /2] was used to calculate the value of the TyG index [18]. For covariates, estimated glomerular filtration (eGFR) was evaluated utilizing the 2009 creatinine equation according to the Chronic Kidney Disease Epidemiology Collaboration. The definition of chronic kidney disease(CKD) was eGFR < 60 mL/min/1.73 m^2^ [19]. Hypertension and dyslipidemia were defined as a self-reported physician diagnosis of a medical condition or being on medication [20].

### Statistical analysis

Continuous variables were applied by mean (standard deviation, SD) or median (interquartile ranges, IQR) depending on normal distribution or not, while the frequency with percentage was presented for categorical variables. All participants were classified by tertiles of the TyG index (T1, T2, T3) according to the TyG index value, and the Kruskal–Wallis test, Chi-square test and ANOVA were conducted to compare differences among various TyG index tertiles as appropriate. Univariable and multivariable logistic regression analyses were conducted to estimate the relationship between DM and CVD for each TyG index group. Model 1 was a rough analysis with no adjustment to evaluate the association between diabetes and new-onset CVD in various tertiles of the TyG index. In Model 2, the analysis was adjusted for age and gender which were the most common confounding biases. In addition, complete adjusted Model 3 was calculated based on further adjustment for BMI, smoking, drinking, education, medical condition (including diabetes, hypertension, dyslipidemia, CKD), laboratory markers (including CRP, uric acid, blood urea nitrogen) and drugs against hypertension and diabetes. *P* for interaction was performed to estimate the tendency in the incrementing value of TyG index cohorts as the substitute of *P* for trend. Subgroup analyses were applied to stratify the relationship between diabetes and CVD for each TyG index group by sex, history of hypertension and dyslipidemia, smoking and drinking. In the sensitivity analysis, the above statistical analyses were performed again by substituting the TyG index, another effective indicator of insulin resistance, TG/HDL-C, to estimate the uniqueness of the trend of the TyG index.

R 4.0.3 (Vienna, Austria) was used for statistical analysis. *P* < 0.05 was regarded as statistically significant (two-sided).

## Results

In total, 6,114 participants in CHARLS were enrolled in this cohort study. The baseline characteristics of all adults in this study based on tertiles of the TyG index value (T1-T3) and the proportion of CVD development are shown in Table 1. The mean value of the TyG index in 2011 was 8.66 (0.65). The prevalence of DM among participants in the T1, T2 and T3 groups was 3.9%, 4.2%, and 4.0%, respectively.

**Table 1:** The baseline characteristics of participants classified by the value of TyG index

	T1	T2	T3	*P *value
N	2038	2038	2038	
TyG index	8.01 (0.25)	8.58 (0.15)	9.38 (0.49)	< 0.001
TG/HDL-C	1.17 (0.45)	2.18 (0.76)	5.98 (5.67)	< 0.001
TG/HDL-C (%)				< 0.001
Q1	1637 (80.3)	385 (18.9)	16 (0.8)	
Q2	396 (19.4)	1305 (64.0)	337 (16.5)	
Q3	5 (0.2)	348 (17.1)	1685 (82.7)	
AGE	57.74 (9.40)	58.15 (8.92)	57.72 (8.61)	0.233
Education (%)				0.027
Low	967 (47.4)	1006 (49.4)	903 (44.3)	
Median	1021 (50.1)	983 (48.2)	1077 (52.8)	
High	50 (2.5)	49 (2.4)	58 (2.8)	
MALE (%)	1034 (50.7)	914 (44.8)	828 (40.6)	< 0.001
BMI	23.70 (4.95)	23.68 (4.91)	23.63 (5.09)	0.911
BUN (mg/dl)	16.17 (4.60)	15.49 (4.22)	15.33 (4.25)	< 0.001
Glucose (mg/dl)	96.49 (13.50)	103.35 (15.63)	126.32 (48.76)	< 0.001
Creatinine (mg/dl)	0.76 (0.18)	0.77 (0.18)	0.78 (0.20)	0.021
TC (mg/dl)	181.32 (32.82)	193.01 (35.92)	205.32 (40.40)	< 0.001
TG (mg/dl)	64.89 (15.04)	106.26 (20.06)	217.51 (118.57)	< 0.001
HDL-C (mg/dl)	59.67 (14.96)	52.43 (13.49)	42.73 (12.16)	< 0.001
LDL-C (mg/dl)	110.54 (28.99)	121.18 (33.00)	116.79 (39.43)	< 0.001
CRP (mg/l)	2.39 (7.52)	2.41 (6.22)	2.52 (6.19)	0.807
Uric Acid (mg/dl)	4.21 (1.12)	4.33 (1.19)	4.62 (1.30)	< 0.001
eGFR	96.7 (87.3, 104.5)	95.7 (86.1, 103.2)	94.3 (83.2, 102.1)	< 0.001
Hypertension (%)	409 (20.1)	363 (17.8)	353 (17.3)	0.054
Dyslipidemia (%)	127 (6.2)	143 (7.0)	123 (6.0)	0.401
Diabetes (%)	81 (4.0)	86 (4.2)	80 (3.9)	0.877
Asthma (%)	63 (3.1)	53 (2.6)	56 (2.7)	0.623
Smoking (%)	774 (38.0)	789 (38.7)	761 (37.3)	0.664
Drinking (%)	679 (33.3)	622 (30.5)	626 (30.7)	0.100
CKD (%)	45(2.2)	52(2.5)	86(4.2)	0.021
Antihypertensive (%)	259 (12.7)	183 (9.0)	188 (9.2)	0.294
Antidiabetic (%)	61 (3.0)	56 (2.7)	54 (2.6)	0.196
CVD in 2018 (%)	239 (11.7)	231 (11.3)	283 (13.9)	0.028

The baseline characteristics of participants with or without DM are shown in Table 2. During the follow-up, 753 participants (12.3%) among 6114 participants had developed new-onset CVD. Among the participants without DM, 700(11.9%) individuals developed CVD during the follow-up, while 53(21.5%) DM participants developed CVD during the follow-up.

**Table 2:** The baseline characteristics of participants classified by DM

	TOTAL	NON-DM	DM	*P *value
N	6114	5867	247	
TyG index	8.66 (0.65)	8.66 (0.65)	8.67 (0.66)	0.735
TyG (%)				0.877
Q1	2038 (33.3)	1957 (33.4)	81 (32.8)	0.877
Q2	2038 (33.3)	1952 (33.3)	86 (34.8)	
Q3	2038 (33.3)	1958 (33.4)	80 (32.4)	
TG/HDL-C	3.11 (3.90)	3.11 (3.92)	3.11 (3.57)	0.992
TG/HDL-C (%)				0.9
Q1	2038 (33.3)	1954 (33.3)	84 (34.0)	
Q2	2038 (33.3)	1954 (33.3)	84 (34.0)	
Q3	2038 (33.3)	1959 (33.4)	79 (32.0)	
Education (%)				0.166
Low	2876 (47.0)	2774 (47.3)	102 (41.3)	
Median	3081 (50.4)	2942 (50.1)	139 (56.3)	
High	157 (2.6)	151 (2.6)	6 (2.4)	
AGE	57.87 (8.98)	57.92 (9.00)	56.78 (8.40)	0.051
Male (%)	2776 (45.4)	2649 (45.2)	127 (51.4)	0.061
BMI	23.67 (4.98)	23.61 (4.94)	25.18 (5.77)	< 0.001
BUN (mg/dl)	15.66 (4.37)	15.65 (4.36)	15.88 (4.56)	0.43
Glucose (mg/dl)	108.72 (33.12)	108.63 (33.00)	110.79 (36.03)	0.316
Creatinine (mg/dl)	0.77 (0.18)	0.77 (0.18)	0.78 (0.21)	0.213
TC (mg/dl)	193.22 (37.80)	193.23 (37.86)	192.98 (36.55)	0.92
TG (mg/dl)	129.55 (95.12)	129.54 (95.10)	129.80 (95.88)	0.967
HDL-C (mg/dl)	51.61 (15.25)	51.61 (15.23)	51.57 (15.80)	0.964
LDL-C (mg/dl)	116.17 (34.35)	116.16 (34.36)	116.36 (34.34)	0.931
CRP (mg/l)	2.44 (6.67)	2.43 (6.66)	2.61 (6.99)	0.673
Uric Acid (mg/dl)	4.39 (1.21)	4.39 (1.22)	4.36 (1.21)	0.69
Hypertension (%)	1125 (18.4)	1023 (17.4)	102 (41.3)	< 0.001
Dyslipidemia (%)	393 (6.4)	319 (5.4)	74 (30.0)	< 0.001
Asthma (%)	172 (2.8)	162 (2.8)	10 (4.0)	0.316
Smoking (%)	2324 (38.0)	2243 (38.2)	81 (32.8)	0.097
Drinking (%)	1927 (31.5)	1860 (31.7)	67 (27.1)	0.148
CVD in 2018 (%)	753 (12.3)	700 (11.9)	53 (21.5)	< 0.001

The TyG index increased the relationship between diabetes and new-onset CVD. Overall, diabetes was positively correlated with CVD risk (OR 1.604, 95% CI 1.357–1.896, *P* < 0.001). After adjusting for age, sex, BUN, history of hypertension, dyslipidemia or CKD, smoking, drinking, BMI, education and medication treatments for diabetes and hypertension, diabetes was positively related to the prevalence of CVD in 2011 (OR 1.475, 95% CI 1.243–1.752, *P* < 0.001) and new-onset CVD risk (OR 1.437, 95% CI 1.032–1.999, *P* = 0.032). There was a gradient increase in the OR for new-onset CVD due to DM across the value of the TyG index based on the completely adjusted model (Table 3, Model 3, *P* for trend < 0.05). The ORs of diabetes for new-onset CVD were 1.657 (95% CI 0.928–2.983, *P* = 0.098), 1.834(95% CI 1.064–3.188, *P* = 0.037) and 2.234(95% CI 1.349–3.673, *P* = 0.006) for T1, T2 and T3, respectively. (Table 3).

**Table 3:** The association between diabetes and cardiovascular disease stratified by TyG index

	T1	*P* value	T2	*P* value	T3	*P* value	*P* for trend
CVD in 2018	239	**/**	231	**/**	283	**/**	**/**
DM in 2015	81	**/**	86	**/**	80	**/**	**/**
Model 1	1.758	0.056	2.001	0.013	2.304	0.001	0.013
Model 2	1.714	0.068	1.974	0.015	2.252	0.002	0.017
Model 3	1.657	0.098	1.834	0.037	2.234	0.006	0.030
Model1	Unadjusted						
Model2	Adjusted for Age, Gender						
Model3	Adjusted for Age, Gender, BMI, smoking, drinking, education, diabetes, hypertension, dyslipidemia, CKD, CRP, UA, BUN and medical treatment (including diabetes and hypertension)						

**Fig. 2:**
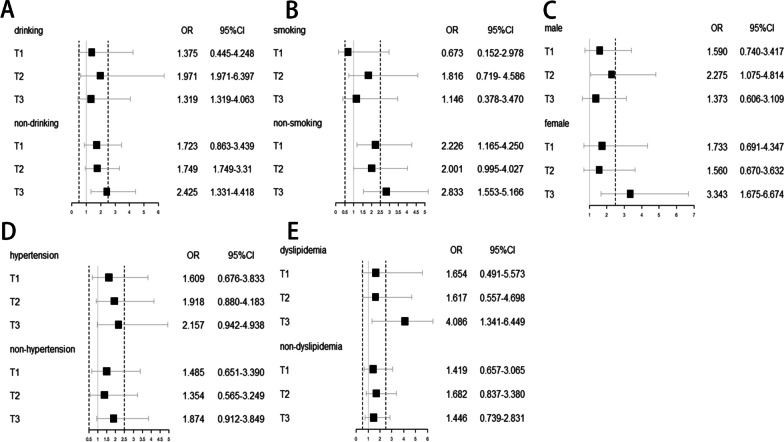
The results of subgroup analyses between diabetes and CVD by TyG index cohorts after adjustment for age, sex, BMI, smoking, drinking, education, diabetes, hypertension, dyslipidemia, CKD, CRP, UA, BUN and medical treatments. Stratification variables were not adjusted in the corresponding models. CI, confidence interval; CVD, cardiovascular disease

Subgroup analyses were utilized to stratify the relationship between diabetes and CVD by potential risk factors. The gradient of increasing risk of CVD still existed among those with hypertension and nondrinkers. The three-way interaction was statistically significant in hypertension (*P* = 0.039) and nondrinkers (*P* = 0.001) (Fig. 2) after complete adjustments. Stratification variables were not adjusted in the corresponding models.

In the sensitivity analysis, there was no apparent gradient of OR of DM in 2011 for new-onset CVD in 2018 across elevated TG/HDL-C values (T1-T3, *P* for trend = 0.312). The ORs of diabetes for CVD in 2018 for T1, T2, and T3 of TG/HDL-C were 1.569 (95% CI 0.876–2.810, *P* = 0.130), 2.195(95% CI 1.283–3.757, *P* = 0.004) and 1.879(95% CI 1.098–3.217, *P* = 0.021) respectively, after total adjustment. (Table 4).

**Table 4:** The association between diabetes and cardiovascular disease stratified by TG/HDL-C

	T1	*P* value	T2	*P* value	T3	*P* value	*P* for trend
Unadjusted	1.731	0.062	2.328	0.002	2.033	0.009	0.334
Model 1	1.680	0.078	2.308	0.002	1.98	0.012	0.315
Model 2	1.569	0.130	2.195	0.004	1.879	0.021	0.312
Model1	Adjusted for Age, Gender						
Model2	Adjusted for Age, Gender, BMI, smoking, drinking, education, diabetes, hypertension, dyslipidemia, CKD, CRP, UA, BUN and medical treatment (including diabetes and hypertension)						

## Discussion

This study explored the effect of the TyG index in predicting future CVD risk in patients with established DM using a longitudinal cohort dataset representing the Chinese population. Even after adjusting for potential confounders, DM was associated with an elevated risk for CVD incidence, with a value of 1.475 (95% CI 1.243–1.752, *P* < 0.001). Most importantly, this study suggested that an increased TyG index aggravates the relationship between diabetes and future CVD.

DM is considered an established risk factor for CVD development [21]. Consistent with prior studies, we confirmed that diabetes was significantly related to increased cardiovascular risk (*P* < 0.001). However, the OR (1.604, 95% CI 1.357–1.896) for cardiovascular disease in adults with DM was lower than those reported in the previous cohort. This disparity is likely driven by differences in race and ethnicity [22–24]. For instance, Asian individuals with diabetes have lower BMI values [25]. A real-world study in China also obtained similar results (HR = 1.747, 95% CI 1.566–1.949 *P* < 0.0001) [26].

IR is a characteristic of diabetes [27, 28], and the TyG index is a critical marker composed of TG and FBG concentrations and a reliable and straightforward alternative to IR [29]. The TyG index was proven to be related to the risk of diabetes in many studies. A retrospective cohort study reported that a higher TyG index has a positive association with an increased incidence of diabetes in Chinese adults older than 45 years, according to the CHARLS data [30]. Another 15-years follow-up study in China showed a nonlinear relationship between the TyG index and typed two diabetes mellitus risks among the general Chinese population, and the cutoff point value was at 8.51 [10]. Compared with fast plasma glucose and triglycerides, a White European study reported that the TyG index was a better predictor of T2DM risk in normoglycemic patients [31]. However, a retrospective study from Thailand demonstrated that the TyG index appeared to be a less robust predictor of diabetes than FPG [32]. In general, the TyG index was regarded as a valuable and independent predictor for the risk of diabetes development.

Subsequently, numerous clinical studies were designed to evaluate the correlation between the TyG index and CVD in general population cohorts with and without diabetes. A Brazilian study that enrolled 2330 patients in secondary care reported that the TyG index positively correlated with high coronary artery disease (CAD) prevalence and atherosclerosis [33]. A prospective cohort study named Kailuan recruited 98,849 participants without myocardial infarction and demonstrated that an increased value of the TyG index at baseline and long-term values independently predicted the incidence of myocardial infarction [34]. Moreover, substantial changes in the TyG index were positively correlated with CVD risk observed in the 62,443 participants without CVD at baseline in China [35]. In addition, in patients who underwent percutaneous coronary intervention (PCI) after acute coronary syndrome, an elevated TyG index was independently related to in-stent restenosis after drug-eluting stent implantation in the 62,443 general population in China with some predictive value [36]. The TyG index was significantly associated with future CVD risk in the current study.

This is the first study in a prospective Chinese population evaluating the possible exacerbation of CVD by a higher TyG index level among participants with DM. The strength of this study is that we applied a sizeable national database representing middle- and older-aged Chinese individuals. Additionally, this study considered and adjusted health lifestyle habits (such as smoking and drinking status) and health status (such as hypertension and CKD) related variables and conducted subgroup analysis. Last, compared with the most recommended imaging diagnostic methods (such as myocardial perfusion scintigraphy) [37], the TyG index may be more cost-effective [38] and have broader potential application scenarios.

The results of our study need to be interpreted in consideration of its limitations. First, some unidentified medications, such as SGLT2 inhibitors, could impact the risk of CVD that could have affected the conclusions. In the current study, TG/HDL-C, another biomarker of IR, was performed in the sensitivity analyses to avoid potential bias and proved the unique trend across the tertiles to the TyG index. Second, our study sample is not ethnically representative because the CHARLS study only enrolled middle-aged to elderly adult populations in China. Future cohorts are needed to expand toward other descent.

## Conclusions

Using a nationally representative sample, this study confirmed that DM is a risk factor for CVD. More importantly, the current study innovatively found that a higher TyG index aggravates the association between DM and the risk of developing CVD. These findings could provide potential clinical guidelines for DM primary prevention of future CVD due to DM. Furthermore, it is also crucial to evaluate the potential benefit of treatment targeting IR in preventing severe complications in the future.

## Data Availability

The data that support the findings of this study are available from the China Health and Retirement Longitudinal Study (CHARLS) but restrictions apply to the availability of these data, which were used under license for the current study, and so are not publicly available. Data are however available from the correspondence authors Jingang Zheng upon reasonable request and with permission of CHARLS.

## Abbreviations

95%CI: 95% Confidence intervals
CVD: Cardiovascular disease
IR: Insulin resistance
TyG: Triglyceride glucose
MACE: Major adverse cardiovascular events
CHARLS: China Health and Retirement Longitudinal Study
TG: Triglycerides
FB: Fast blood glucose
TC: Total cholesterol
hsCRP: High-sensitivity C-reactive protein
HDL-C: High-density lipoprotein cholesterol
LDL-C: Low-density lipoprotein cholesterol
BUN: Blood urea nitrogen
DM: Diabetes mellitus
eGFR: Estimated glomerular filtration
CKD: Chronic kidney disease
IQR: Interquartile ranges
SD: Standard deviation
CAD: Coronary artery diseases
PCI: Percutaneous coronary intervention
